# Methods to Crosswalk Between Cognitive Test Scores Using Data From the Alzheimer’s Disease Neuroimaging Cohort

**DOI:** 10.1093/geroni/igaf122.1855

**Published:** 2025-12-31

**Authors:** Sarah Ackley, Jingxuan Wang, Ruijia Chen, Tanisha Hill-Jarrett, L Paloma Rojas-Saunero, Andrew Stokes, Sachin Shah, M Maria Glymour

**Affiliations:** Brown University, Providence, Rhode Island, United States; University of California, San Francisco, San Francisco, California, United States; Boston University School of Public Health, Boston, Massachusetts, United States; University of California Memory and Aging Center, San Francisco, California, United States; University of California, Los Angeles, Los Angeles, California, United States; Boston University, Boston, Massachusetts, United States; Massachusetts General Hospital, Boston, Massachusetts, United States; Boston University, Boston, Massachusetts, United States

## Abstract

Studies use multiple instruments to measure dementia-related outcomes, making comparisons of intervention or exposure effects challenging. For example, trials of anti-amyloid drugs like TRAILBLAZER-AD 2 (donanemab), CLARITY-AD (lecanemab), and GRADUATE I & II (gantenerumab) report changes in the clinical dementia rating sum of boxes (CDR-SB) as the primary outcome, while earlier trials used measures such as the Mini-Mental State Exam (MMSE) or the Alzheimer’s Disease Assessment Scale-Cognitive Subscale. Comparing or meta-analyzing findings requires integrating effect estimates from different outcome measures. Psychometric methods can harmonize these measures but rely on unverifiable assumptions and do not work with summary statistics. To address this gap, we developed two methods to crosswalk estimated treatment effects on cognitive outcomes that are flexible, broadly applicable, and do not rely on strong distributional assumptions. We present two methods to crosswalk effect estimates using one measure to estimates using another measure, illustrated with global cognitive measures from the Alzheimer’s Disease Neuroimaging Initiative (ADNI). Specifically, we develop crosswalks for the following measures and associated change scores over time: the clinical dementia rating scale sum of box (CDR-SB), Montreal cognitive assessment (MoCA), and mini-mental state exam (MMSE) scores. Finally, a setting in which crosswalking is not appropriate is illustrated with plasma phosphorylated tau (p-tau) concentration and global cognitive measures. Given the inconsistent collection and reporting of dementia and cognitive outcomes across studies, these crosswalking methods offer a valuable approach to harmonizing and comparing results reported on different scales.

